# Peri-Operative Pain and Opioid Use in Opioid-Naïve Patients Following Inpatient Head and Neck Surgery

**DOI:** 10.3389/fpsyt.2022.857083

**Published:** 2022-07-08

**Authors:** Danielle R. Trakimas, Carlos Perez-Heydrich, Rajarsi Mandal, Marietta Tan, Christine G. Gourin, Carole Fakhry, Wayne M. Koch, Jonathon O. Russell, Ralph P. Tufano, David W. Eisele, Peter S. Vosler

**Affiliations:** Department of Otolaryngology – Head and Neck Surgery, Johns Hopkins Hospital, Baltimore, MD, United States

**Keywords:** head and neck surgery, post-operative analgesia, opioids, prescriptions, pain

## Abstract

Pain management is an important consideration for Head and Neck Cancer (HNC) patients as they are at an increased risk of developing chronic opioid use, which can negatively impact both quality of life and survival outcomes. This retrospective cohort study aimed to evaluate pain, opioid use and opioid prescriptions following HNC surgery. Participants included patients undergoing resection of a head and neck tumor from 2019–2020 at a single academic center with a length of admission (LOA) of at least 24 h. Exclusion criteria were a history of chronic pain, substance-use disorder, inability to tolerate multimodal analgesia or a significant post-operative complication. Subjects were compared by primary surgical site: Neck (neck dissection, thyroidectomy or parotidectomy), Mucosal (resection of tumor of upper aerodigestive tract, excluding oropharynx), Oropharyngeal (OP) and Free flap (FF). Average daily pain and total daily opioid consumption (as morphine milligram equivalents, MME) and quantity of opioids prescribed at discharge were compared. A total of 216 patients met criteria. Pain severity and daily opioid consumption were comparable across groups on post-operative day 1, but both metrics were significantly greater in the OP group on the day prior to discharge (DpDC) (5.6 (1.9–8.6), *p* < 0.05; 49 ± 44 MME/day, *p* < 0.01). The quantity of opioids prescribed at discharge was associated with opioid consumption on the DpDC only in the Mucosal and FF groups, which had longer LOA (6–7 days) than the Neck and OP groups (1 day, *p* < 0.001). Overall, 65% of patients required at least one dose of an opioid on the DpDC, yet 76% of patients received a prescription for an opioid medication at discharge. A longer LOA (aOR = 0.82, 95% CI: 0.63–0.98) and higher Charlson Comorbidity Index (aOR = 0.08, 95% CI: 0.01–0.48) were negatively associated with receiving an opioid prescription at the time of discharge despite no opioid use on the DpDC, respectively. HNC patients, particularly those with shorter LOA, may be prescribed opioids in excess of their post-operative needs, highlighting the need the for improved pain management algorithms in this patient population. Future work aims to use prospective surveys to better define post-operative and outpatient pain and opioid requirements following HNC surgery.

## Introduction

Prescription opioids have significantly contributed to the recent opioid epidemic. Multiple studies across surgical specialties have shown that patients are prescribed opioids in excess of their post-operative requirements (1, 2). With excess medication available, patients are more likely to use opioids for prolonged periods of time, thereby increasing the risk for chronic opioid use (3). Additionally, unused medication has the potential for diversion (1). Recent data has shown that death rates from synthetic opioids, including prescription opioids, have steeply risen since 2013 (4), further highlighting the need to limit prescription of these medications.

Pain management is an important consideration for quality of life as well as survival outcomes in Head and Neck Cancer (HNC) patients (5–8). These patients often require surgical treatment that leads to significant peri-operative pain and disfigurement, and up to 50% (9) also suffer from psychiatric comorbidities (10, 11). These factors increase the risk of opioid dependence, and it is estimated that between 20–60% of HNC patients develop chronic opioid use after treatment (7, 12–14). This is particularly important, as chronic opioid use following surgery for HNC has been associated with decreased disease-free survival (15).

Recent efforts have been made across the United States to limit inappropriate prescribing of opioids, and clinical practice guidelines have been developed to improve management of pain in the perioperative setting (16–18). Within the Otolaryngology literature, studies have focused on evaluating various multimodal analgesic regimens for post-operative pain management following tonsillectomy (19), as it's one of the most painful surgical procedures (20, 21), and endoscopic sinus surgery (3, 22), given the high volume of cases. However, there are limited data on peri-operative pain management for complex HNC patients following inpatient procedures.

To provide safer and more effective pain management guidelines for HNC patients, it is essential to better define post-operative pain and opioid requirements in this patient population. Herein we evaluate patterns of pain, opioid use, and opioid prescriptions following inpatient surgeries for HNC patients and highlight the need for improved pain management algorithms in this patient population.

## Materials and Methods

### Patients

**Figure 1:**
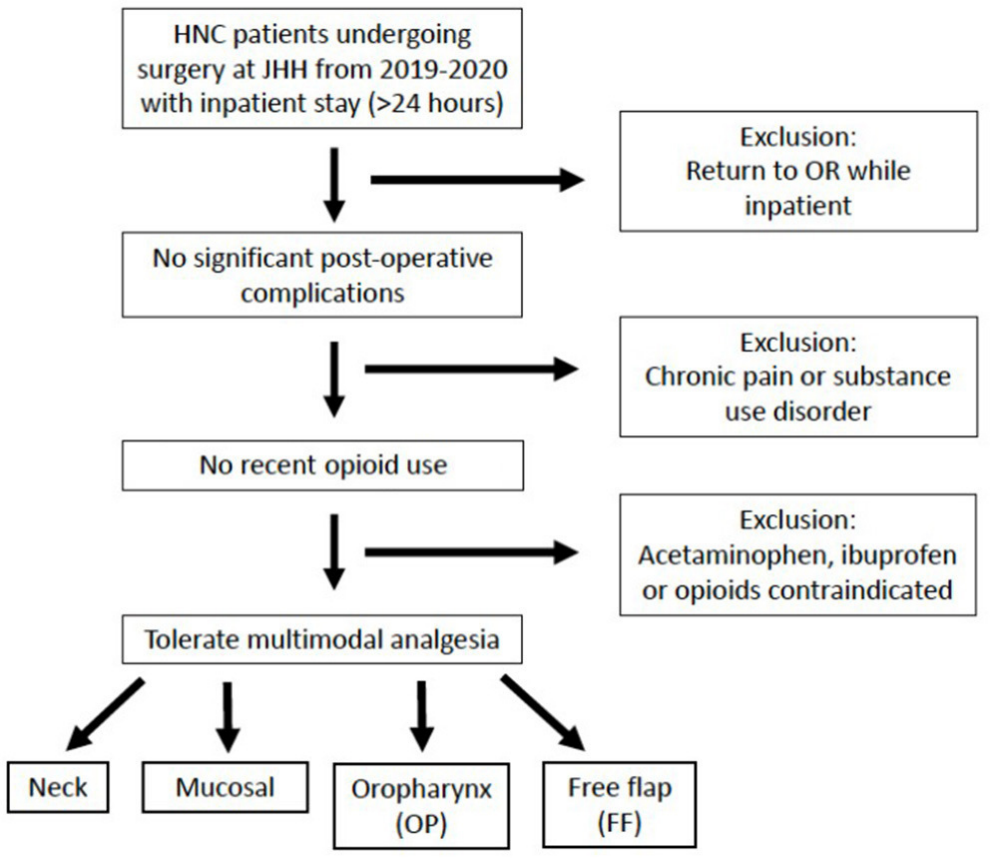
Inclusion and exclusion criteria. Patients undergoing surgery for a head and neck primary tumor at Johns Hopkins Hospital (JHH) from 2019–2020 were included if they had no significant post-operative complications, no recent opioid use and could tolerate multimodal analgesia. Patients were categorized into four groups based on their surgical procedure. HNC, head and neck cancer; OR, operating room.

A retrospective review of patients undergoing surgery for tumors of the head and neck in the Otolaryngology – Head & Neck Surgery department was performed at a single academic institution. This project was reviewed and approved by our institutional review board (IRB00251111). Inclusion criteria were: (1) age 18 years or older, (2) surgery for tumor in the head and neck region, (3) surgery from January 1, 2019 – January 1, 2020 at Johns Hopkins Hospital, (4) length of admission (LOA) following surgery of at least 24 h (measured from the time the patient was awake and recovered from anesthesia) and (5) “opioid naïve,” defined as no opioid consumption within the 30 days prior to surgery. Exclusion criteria were: (1) past medical history of chronic pain or substance use disorder, (2) inability to tolerate multimodal analgesia (i.e., scheduled acetaminophen and ibuprofen and as needed opioids) due to medical comorbidity (specifically severe hepatic or renal impairment, cirrhosis or moderate-severe chronic kidney disease) or intolerance or allergy and (3) post-operative complication requiring return to the operating room during admission. Patients were grouped by surgical procedure as follows: (1) Neck group: neck dissection (ND) alone or thyroidectomy or parotidectomy with or without ND), (2) Mucosal group: resection of tumor of the upper aerodigestive tract other than in the oropharynx, with or without ND, but without free flap reconstruction (3) Oropharyngeal (OP) group: resection of primary oropharyngeal tumor with or without ND, but without free flap reconstruction, (4) Free flap (FF) group: resection of tumor of upper aerodigestive tract requiring reconstruction with a free flap (Figure 1).

### Data Collection and Analysis

Patient demographics, including age, sex and ethnicity, and past medical and surgical history were collected by chart review from our electronic medical record (EMR) system. Charlson Comorbidity Index (CCI) scores were calculated based on ICD-9 codes (23, 24). Cancer staging was reported according to the American Joint Committee of Cancer 8th Edition Cancer Staging Manual (25). Operative notes for the patients' surgery of interest were manually reviewed and categorized into the one of the 4 surgical groups listed above. Additional operative notes during the patients' admission were reviewed to identify post-operative complications that required return to the operating room, including hematoma/seroma, abscess, fistula or free flap concerns. Pain while inpatient was recorded on a Likert scale from 0 (no pain) to 10 (worst pain) by nursing staff, and daily average pain was calculated. Daily opioid consumption while inpatient and total quantity of opioids prescribed at discharge from the hospital were calculated and converted to morphine milligram equivalents (MME) (26). Inpatient-use of benzodiazepines, Z-drugs (i.e., zaleplon, zolpidem or eszopiclone), serotonin reuptake inhibitors (SRIs) and gabapentinoids (gabapentin or pregabalin) were also recorded.

### Statistical Analysis

Continuous variables are reported as the median and range or mean and standard deviation (SD), where applicable. Kruskal-Wallis test was used to compare continuous measurements between multiple groups, and Fisher's exact and Chi-squared tests were used to compare categorical variables between two and more than two groups, respectively. Wilcoxon matched-pairs signed rank test was used to compare paired data from individual patients at different time points. Linear regression analysis was used to determine significant predictors of quantity of opioids consumed following surgery and quantity of opioids prescribed at discharge; this was performed for all patients as well as within each surgical group. Multivariable logistic regression analysis was used to evaluate factors associated with receiving an opioid prescription at the time of discharge in patients with no opioid use during the 24 h prior to discharge (DpDC). Independent variables evaluated include age, sex, race, cancer stage, CCI, surgical procedure, LOA, and average pain severity on the DpDC. Of note, the surgical procedure group OP was not included as an independent variable given the small number of OP patients in the subgroup evaluated in this regression analysis. Results are reported as the adjusted odds ratio (aOR) and 95% confidence interval (CI). All statistical analysis was performed using R Statistical Software (version 4.1.0; R Foundation for Statistical Computing, Vienna, Austria).

## Results

A total of 216 patients met inclusion criteria. Demographics and clinical information about the patient cohort are shown in Table 1. The Neck group had a significantly lower median age at the time of surgery (52 (18–89) years) compared to the Mucosal (67 (38–86) years, *p* < 0.001) and FF (61 (42–80) years, *p* < 0.001) groups. The proportion of females was significantly greater in the Neck group (68%) compared to all others, which averaged between 25-31% female (*p* < 0.001). The CCI was significantly lower in the Neck group (2–15) compared to the Mucosal (8 (2–19), *p* < 0.001) and FF (6 (2–15), *p* < 0.05) groups. Considering tumors able to be staged, the Neck and OP groups had a significantly higher proportion of Stage I or II tumors compared to the Mucosal and FF groups (*p* < 0.001). Similarly, a significantly lower proportion of patients had a history of radiation therapy (RT) in the Neck group (1%) compared to the Mucosal (18%, *p* < 0.01) and FF (20%, *p* < 0.01) groups. The median LOA was approximately 1 day for the Neck and OP groups, compared to 6–7 days for the Mucosal and FF groups (*p* < 0.001). Overall use of benzodiazepines and Z-drugs was low, 6% and 1%, respectively, and did not significantly differ between groups. A greater proportion of patients used SRIs (18%) and gabapentinoids (13%) while inpatient, with a significantly lower proportion of gabapentinoid-use in the Neck (5%) group compared to Mucosal (18%, *p* < 0.01) and FF (26%, *p* < 0.01) groups.

**Table 1:** Patient demographics and clinical information.

**Variable** **Median (range)**	**Neck**	**Mucosal**	**OP**	**FF**	**Total**
*N*	108	45	20	43	216
Procedure, *N*	ND (20) Parotid ± ND (23) Thyroid ± ND (65)	OC ± ND (26) TL ± ND (19)	TORS ± ND (20)	Scapula (1) Fibula (10) RF (15) ALT (17)	
Age (years)	52*** (18–89)	67 (38–86)	58 (48–75)	61 (42–80)	60 (18–89)
Female, *N* (%)	73 (68)	14 (31)	5 (25)	13 (30)	105 (49)
Caucasian, *N* (%)	72 (67)	29 (64)	18 (90)	30 (70)	149 (69)
CCI	4 (2–15)***	8 (2–19)	5 (2–10)	6 (2–15)	6 (2–19)
Cancer stage, *N* (%)	I/II: 42 (39)*** III/IV: 1 (1) NA: 65 (60)	I/II: 7 (16) III/IV: 26 (58) NA: 12 (26)	I/II: 20 (100)*** III/IV: – NA: –	I/II: 13 (30) III/IV: 21 (49) NA: 9 (21)	I/II: 82 (38) III/IV: 48 (22) NA: 86 (40)
Prior RT, *N* (%)	1 (1)**	8 (18)	–	8 (19)	17 (8)
LOA (days)	1*** (1–6)	6* (1–19)	1*** (1–5)	7 (2–22)	1 (1–22)
Medications, *N* (%)					
Benzodiazepine: Z–drug: SRI: Gabapentinoid:	2 (2) 1 (1) 19 (18) 5 (5)**	4 (9) 2 (4) 7 (16) 8 (18)	2 (10) 0 (0) 5 (25) 3 (15)	4 (9) 0 (0) 8 (19) 11 (26)	12 (6) 3 (1) 39 (18) 27 (13)
Pain service consult, *N* (%)	0 (0)	4 (9)	0 (0)	4 (9)	8 (4)

ALT, anterolateral thigh; CCI, Charlson Comorbidity Index; F, female; FF, free flap reconstruction; LOA, length of admission; N, number; NA: not applicable; ND, neck dissection; OC, oral cavity; OP, oropharynx; RF, radial forearm; Rsn, resection; RT, radiation therapy; SD, standard deviation; SRI: serotonin reuptake inhibitor; TORS, transoral robotic surgery; TL, total laryngectomy. *p < 0.05, **p < 0.01, ***p < 0.001.

### Overall Pain and Opioid Consumption While Inpatient

Average daily pain and total daily opioid consumption were compared between surgical groups on the first post-operative day (POD 1) and the day prior to discharge from the hospital (DpDC) (Table 2). Pain severity on POD 1 tended to be highest in the OP group (4.9 ± 2.1) compared to the Neck (3.8 ± 1.6), Mucosal (4.2 ± 2.2) and FF groups (3.9 ± 2.7), but this was not statistically significant. On the DpDC, pain severity remained elevated in the OP group (5.6 (1.9–8.6)), which was significantly greater than in the Neck (3.6 ± 1.9, *p* < 0.01), Mucosal (3.8 ± 2.4, *p* < 0.05), and FF (3.5 ± 1.7, *p* < 0.01) groups. On POD 1, 86% of all patients required at least one dose of an opioid medication for pain control. By the DpDC, only 65% of all patients consumed at least 1 dose of an opioid medication. Average daily opioid consumption on the DpDC was significantly greater in the OP group (49 ± 44 MME/day) compared to the Neck (23 ± 25 MME/day, *p* < 0.01) and FF (25 ± 29 MME/day, *p* < 0.01) groups.

**Table 2:** Average pain and opioid consumption on the first day after surgery and the day prior to discharge from the hospital.

	**Neck**	**Mucosal**	**OP**	**FF**	**Total**
**Pain severity: mean (SD)**					
**POD1:**	3.8 (1.6)	4.2 (2.2)	4.9 (2.1)	3.9 (2.7)	4.0 (2.0)
**DpDC:**	3.6 (1.9)	3.8 (2.4)	5.3 (1.9)**	3.5 (1.7)	3.8 (2.0)
**Opioid consumption and prescriptions: mean (SD)**					
**POD1:**					
MME/day 5mg-Oxy/day	28 (24) 4 (3)	42 (41) 6 (6)	48 (40) 6 (5)	44 (45) 6 (6)	34 (33) 10 (10)
**DpDC:**					
MME/day 5mg-Oxy/day	23 (25) 3 (3)	39 (58) 5 (7)	49 (44)** 7 (6)	25 (29) 3 (4)	27 (32) 8 (10)
MME>0 on DpDC, *N* (%):	69 (64)	27 (60)	18 (90)	27 (63)	141 (65)
**Prescribed at discharge:**					
Total MME Total 5mg-Oxy	118 (73)* 16 (10)	161 (202) 21 (27)	450 (274)*** 60 (37)	216 (240) 29 (32)	174 (190) 52 (57)
Opioid Rx on DC, *N* (%):	85 (78)	29 (64)	18 (90)	32 (74)	164 (76)

5mg-Oxy/day, number of 5mg oxycodone tablets/day; DC, discharge; DpDC, day prior to discharge; FF, free flap reconstruction; MME, morphine milligram equivalents; N, number; OP, oropharynx; POD 1, postoperative day 1; Rx, prescription; SD, standard deviation. *p < 0.05, **p < 0.01, ***p < 0.001.

Linear regression analysis showed a weak association of pain levels with the quantity of opioids consumed on POD1 in the Neck (R^2^ = 0.49, p < 0.001) and OP (R^2^ = 0.44, *p* < 0.01) groups, and on the DpDC within the Neck group (R^2^ = 0.45, p <0.001). Older age was weakly associated with lower opioid consumption within the FF group on the DpDC (R^2^ = 0.31, *p* < 0.001). Additionally, subgroup analysis showed significantly higher average pain levels and daily opioid consumption in the subgroup of all patients taking SRIs (*N* = 39) versus those not taking SRIs (*N* = 177) on POD 1 (5.0 ± 1.2 vs. 3.8 ± 2.0, *p* < 0.01; 46 ± 33 vs. 34 ± 35 MME/day, *p* < 0.01, respectively) and the DpDC (4.4 ± 2.3 vs. 3.6 ± 1.9, *p* < 0.05; 46 ± 44 vs. 26 ± 35 MME/day, *p* < 0.001, respectively). Similarly, the subgroup of all patients taking gabapentinoids (*N* = 27) while inpatient had higher average pain levels than those not taking gabapentinoids (*N* = 189) on POD1 (5.0 ± 2.1 vs. 3.9 ± 2.0, *p* < 0.05) and the DpDC (4.8 ± 2.2 vs. 3.6 ± 2.0, *p* < 0.01); with no difference in opioid consumption between subgroups. Otherwise, sex, ethnicity, CCI score, cancer stage, history of RT and LOA were not associated with average opioid consumption within each surgical group or overall.

### Opioid Prescriptions at the Time of Discharge

Overall, 76% of patients received a prescription for an opioid medication at the time of discharge. The quantity of opioids prescribed at the time of discharge was compared between surgical groups, as well as to average daily pain and total daily opioid consumption on the DpDC within each group. Similar to opioid consumption on the DpDC, the OP group was prescribed the greatest quantity of opioids at discharge (450 ± 274 MME); this was 2–3 times greater than the quantity prescribed to the Neck (118 ± 73 MME, *p* < 0.001), Mucosal (161 ± 202 MME, *p* < 0.001) and FF groups (216 ± 240 MME, *p* < 0.001). Additional subgroup analysis showed no difference in the quantity of opioids prescribed at discharge between subgroups of all patients taking SRIs versus those not taking SRIs while inpatient; with similar findings for subgroups based on gabapentinoid-use while inpatient. Multivariable logistic regression analysis was used to evaluate factors associated with receiving an opioid prescription at the time of discharge in patients with no opioid consumption during the 24 h prior to discharge (Table 3). Within this subset of patients, CCI (aOR = 0.08, 95% CI: 0.01–0.48) and LOA (aOR= 0.82, 95% CI: 0.63–0.98) were associated with a lower incidence of receiving an opioid prescription despite no consumption on the DpDC.

**Table 3:** Odds of receiving an opioid prescription on discharge in patients with no opioid consumption on the day prior to discharge (DpDC).

**Variable**	**aOR**	**95% CI**
Age (>60 years)	1.54	(0.27–10.8)
Sex (Female)	0.62	(0.09–3.89)
Race (Caucasian)	1.59	(0.23–12.35)
CCI (>6)	**0.08**	**(0.01–0.48)**
Cancer stage (III/IV)	1.25	(0.16–12.66)
Procedure:		
Neck FF	3.92 8.46	(0.34–63.9) (1.04–103.47)
LOA (days)	**0.82**	**(0.63–0.98)**
Pain on DpDC (>3)	0.58	(0.11–2.88)

*aOR, adjusted odds ratio; CCI, Charlson Comorbidity Index; CI, confidence interval; DpDC, day prior to discharge; FF, free flap reconstruction; LOA, length of admission*.

*Bold values indicate statistical significance*.

### Trends in Pain and Opioid Consumption

**Figure 2:**
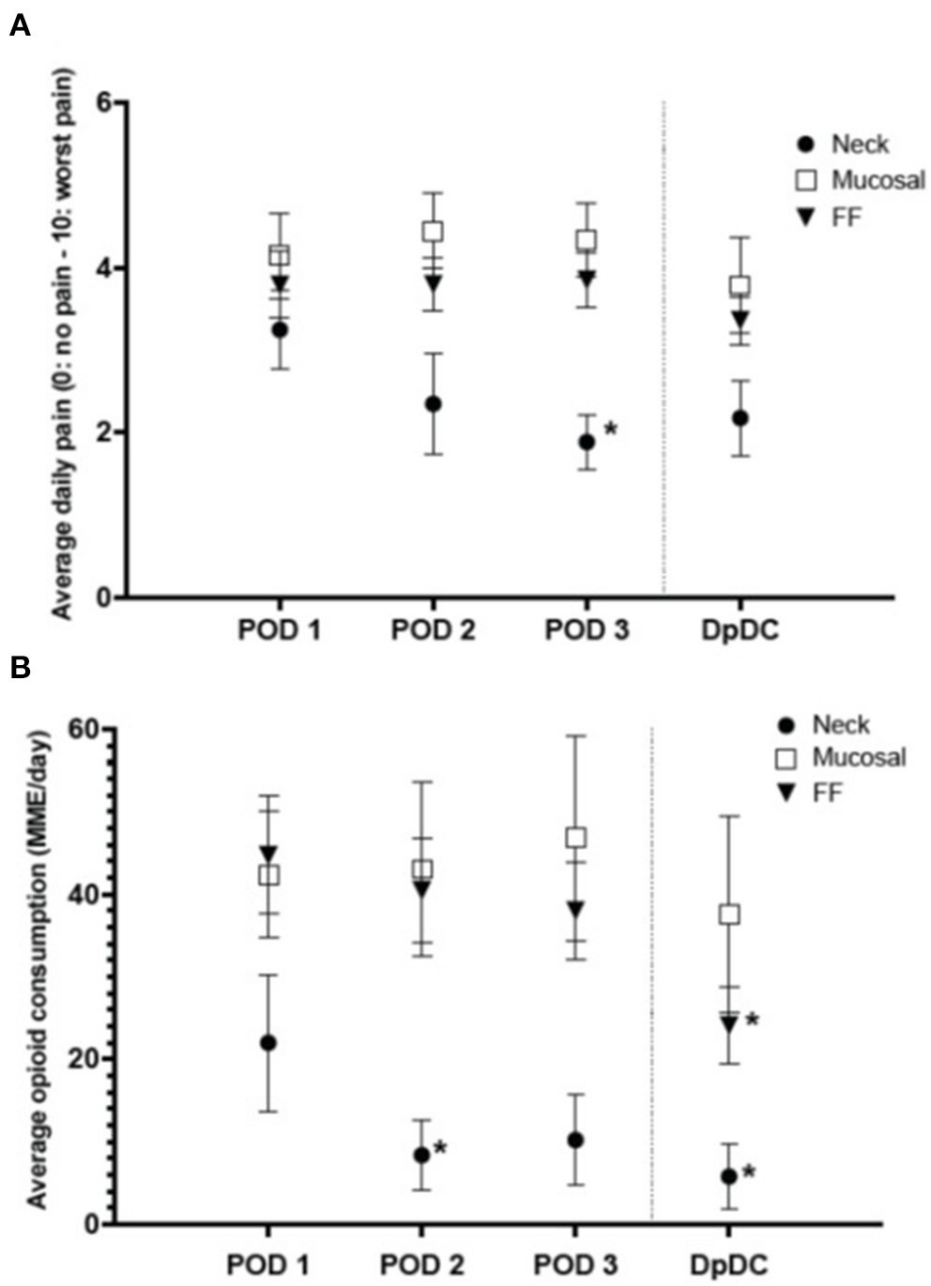
Average daily pain and opioid consumption in a subset of patients with length of admission of 3 or more days (mean ± SEM). **(A)** Average daily pain significantly decreased from post-operative day 1 (POD 1) to POD 3 in the neck group (*p* < 0.05). **(B)** Daily opioid consumption significantly decreased from POD 1 to POD 2 in the neck group (*p < 0.05) and from POD 1 to the day prior to discharge (DpDC) in the free flap (FF) group (*p < 0.05). MME, morphine milligram equivalents.

Patients with length of admission of 3 days or longer were evaluated to determine trends in pain and opioid consumption over time (Figure 2). The Neck group showed a significant decrease in baseline pain from POD 1 to POD 3 (3.3 ± 1.3 to 1.8 ± 0.9, *p* < 0.05) (Figure 2A) and baseline opioid consumption from POD 1 to POD 2 (22 ± 26 MME/day to 8 ± 13 MME/day, *p* < 0.05) (Figure 2B), which remained stable until discharge. All other groups had no change from baseline pain or opioid consumption through POD 3.

## Discussion

This study analyzes post-operative pain and opioid consumption in a cohort of patients undergoing resection of tumors of the head and neck. Overall, we found that most patients (86%) consumed at least one dose of opioid medication on the first post-operative day. By the day prior to discharge, this had decreased to only 65% of all patients, and only 60% of patients had substantial opioid requirements of 7.5 MME/day (5 mg oxycodone/day). Despite these modest numbers, 76% of all patients received an opioid prescription at discharge. There was also discrepancy in the quantity of opioids prescribed between groups with similar pain levels as demonstrated by the FF group receiving nearly twice as much opioid than the Neck group. While the length of admission varied between patients, all patients had at least 24 h of data of pain levels and opioid consumption prior to discharge.

The American Academy of Otolaryngology recently published a comprehensive Clinical Practice Guideline providing evidence-based recommendations for opioid prescribing after common Otolaryngology procedures (18). These guidelines include expected average durations of pain after many Otolaryngology procedures; patients undergoing “Neck” procedures, such as thyroidectomy or parotidectomy, are expected to have pain for up to 3–5 days after surgery (18). In agreement with prior studies referenced in these guidelines, the Neck group in our study had a significant decrease in both pain and opioid consumption during the first three post-operative days, in a subset of patients with at least 3 days of admission. From POD 1 to 2, average opioid consumption in this subset decreased by over 50% from 22 MME/day to less than 10 MME/day, suggesting the majority of patients undergoing these procedures may require only a short supply of or even no opioid medication following surgery. Despite these modest numbers, patients in the Neck group were prescribed an average of 118 MME (equivalent to 16 5-mg tablets of oxycodone) on discharge, clearly in excess of this group's opioid requirements. While a patient's opioid consumption prior to discharge can be used to estimate requirements as an outpatient (17), the majority of patients undergoing Neck procedures are discharged on POD 1 or 2; as such, this group may be over-prescribed opioids on discharge if based on their inpatient requirements during the first 24 h after surgery. Unfortunately, there is limited information in the literature about expected durations of pain and opioid consumption after more complex HNC procedures.

Pain severity did not strongly correlate with opioid consumption or quantity of opioid prescribed at discharge overall or within each surgical group. However, the Mucosal and FF groups had a moderate association between opioid consumption on the day prior to discharge and the quantity of opioids prescribed at discharge. Both groups also had longer lengths of admission, of 6–7 days, compared to 1 day in the Neck and OP groups. This further suggests that prescribing patterns may better represent inpatient opioid requirements as more longitudinal data about patients is available.

Numerous efforts have been made to further our understanding of factors affecting post-operative pain since the role of prescription opioids in the opioid epidemic has been elucidated (19–21, 27). Guidelines for identifying patients at risk for opioid dependence have also been developed (28, 29). We found that patients taking SRIs had significantly higher pain levels and greater consumption of opioids following surgery than those not taking SRIs. This supports other literature that links psychiatric comorbidities of anxiety and depression with post-operative opioid use and incidence of chronic pain (2, 30, 31) and highlights the importance of more effective and safer alternatives specifically for this patient population.

Multimodal analgesia with NSAIDs has become the standard of care following most Otolaryngology procedures, including tonsillectomy, since studies have shown no increased risk of bleeding (32); the use of adjunctive medications for pain, such as gabapentinoids, has also increased in recent years (33). Within our cohort, patients undergoing more extensive surgeries or with higher levels of post-operative pain were more likely to be prescribed a gabapentinoid in addition to standard multimodal analgesia, but there was no difference in daily opioid consumption based on gabapentinoid-use. Multiple studies have also shown an analgesic effect of placebo treatment, yet there remains controversy over the general public's perception of placebo, with regards to deception (34). However, recent data from open-label trials suggest that placebo may still be effective without the need to withhold information from patients; suggesting this may be a feasible addition to standard multimodal analgesia regimens in the future (35). Acute pain and palliative care specialists also play an important role in the post-operative setting, particularly for more complex cases. Only a small proportion of patients in our cohort, 4%, received a pain consult following surgery. However, as options for adjunctive pain management continue to expand, these specialties may become essential in providing safer and more effective post-operative pain regimens for patients.

Despite the aforementioned progress, opioid medications continue to be prescribed in excess of patients' needs following tonsillectomy (21) and other common Otolaryngology surgeries (2, 3), with similar findings in other surgical fields (1, 17, 36). Our data is limited without information about opioid consumption following discharge from the hospital. However, comparison of opioid prescriptions to consumption while inpatient suggests many of these patients were prescribed excess medication, particularly those who received a prescription despite no documented opioid consumption for the 24 h prior to discharge. We found that patients with the highest risk of receiving a prescription despite no use on the DpDC were those with less comorbidities and shorter durations of admission. While providers may be more cautious to prescribe opioids to patients with more comorbidities, given the risk of side effects, the same diligence should be maintained with all patients.

The above findings highlight potential to improve our estimation of patients' opioid requirements following discharge from the hospital. Recent efforts by a General Surgery group showed that in patients undergoing inpatient general surgery procedures, opioid consumption on the day prior to discharge was correlated with outpatient use, suggesting this information could be used as a metric for prescribing (17). Follow-up studies from this group also found that educating surgeons on guidelines for opioid prescribing significantly decreased the amount of opioids prescribed at their institution without increasing patient requests for refill medications (1, 36). In the context of HNC patients, pre-operative chronic pain and chronic opioid use increase a patient's expected opioid needs following surgery (12, 13, 15). This study focused on HNC patients without pre-operative chronic pain or recent opioid use to evaluate post-operative pain following HNC surgery without these additional variables. In our patient cohort the quantity of opioids prescribed correlated with opioid consumption prior to discharge for patients with the longest durations of admission. This was not the case for patients with shorter admissions, suggesting further information, such as data about opioid use after discharge from the hospital, is required to prevent over-prescribing in these patients. While this is feasible, it's important to note that such data is inherently limited by selection bias for those who agree to participate and accuracy of reporting by patients.

This study is limited by the relatively small sample size and retrospective nature of the EMR review. As guidelines have been implemented to limit the quantity of opioids physicians can initially prescribe, we aimed to evaluate data from a recent and limited time period for consistency. Patients with specific comorbidities preventing the use of multimodal analgesia with scheduled acetaminophen and ibuprofen were excluded to compare a more homogenous population, which may limit external validity. While total quantity of opioids prescribed at discharge may be influenced by concern about access to refills, this is less likely as all prescribers at our institution are able to electronically prescribe scheduled medications and an on-call physician is available around the clock for patient phone calls. Finally, this current study is limited to the evaluation of inpatient data.

Effective and safe pain management following HNC surgery is imperative for both quality of life and survival outcomes in this patient population. Despite recent progress in decreasing prescription opioids, our study shows that these medications continue to be prescribed in excess of patients' post-operative needs. Future work aims to better define post-operative pain and opioid requirements following HNC surgery. Our group is currently collecting data on daily opioid consumption after discharge from the hospital with prospective surveys in a comparable patient population. This information will allow us to investigate patient and surgical factors, such as opioid consumption on the day before discharge from the hospital or surgical procedure, associated with outpatient opioid consumption. The ultimate goal of these studies is to develop a model to improve our prediction of a patient's opioid requirements, thereby limiting the risk associated with excess opioid prescriptions.

## Data Availability Statement

The raw data supporting the conclusions of this article will be made available by the authors, without undue reservation.

## Ethics Statement

The studies involving human participants were reviewed and approved by Johns Hopkins IRB. Written informed consent for participation was not required for this study in accordance with the national legislation and the institutional requirements.

## Author Contributions

DT, CP-H, and PV contributed to conception and design of the study. DT and CP-H organized the database and performed the statistical analysis. DT wrote the first draft of the manuscript. All authors contributed to the article and approved the submitted version.

## Funding

This research was supported by NIH T32DC000027-32 grant.

## Conflict of Interest

The authors declare that the research was conducted in the absence of any commercial or financial relationships that could be construed as a potential conflict of interest.

## Publisher's Note

All claims expressed in this article are solely those of the authors and do not necessarily represent those of their affiliated organizations, or those of the publisher, the editors and the reviewers. Any product that may be evaluated in this article, or claim that may be made by its manufacturer, is not guaranteed or endorsed by the publisher.
